# An invertebrate NLR recognizes viral nucleic acids and balances the antiviral signaling pathway through interaction with STING and Cyclophilin A

**DOI:** 10.1371/journal.ppat.1013433

**Published:** 2025-08-18

**Authors:** Shihao Li, Xuechun Li, Mingzhe Sun, Fuhua Li

**Affiliations:** 1 State Key Laboratory of Breeding Biotechnology and Sustainable Aquaculture, Institute of Oceanology, Chinese Academy of Sciences, Qingdao, China; 2 Laboratory for Marine Biology and Biotechnology, Qingdao Marine Science and Technology Center, Qingdao, China; Tulane University School of Medicine, UNITED STATES OF AMERICA

## Abstract

Intracellular recognition of viral nucleic acids by NLRs and subsequent activation of antiviral immunity are crucial for host defense against virus infection in vertebrates. However, understanding on these processes is very limited in invertebrates, especially for crustaceans. In the present study, an NLR gene belonging to the NLRC subfamily (*LvNLRC*) was identified in the Pacific whiteleg shrimp *Litopenaeus vannamei* and its functions in intracellular recognition to DNA virus and antiviral immunity during WSSV infection were elucidated. LvNLRC possesses an ability to detect DNA viral mimics such as poly(dA:dT) with a dose-dependent manner through its leucine-rich repeat (LRR) domain. The LRRs domain also recognizes the partial DNA encoding VP24 of WSSV. LvNLRC could modulate the interferon system-like antiviral response in shrimp through direct interaction between its NACHT domain and LvSTING. Different from the regulatory mechanism in vertebrates, the presence of poly(dA:dT) does not affect the release of STING from the NACHT domain of LvNLRC in shrimp. Interestingly, shrimp Cyclophilin A (LvCypA) can directly interact with the NACHT domain of LvNLRC in a dose-dependent manner in the presence of poly(dA:dT). Furthermore, LvCypA also plays an important role in the regulation of the interferon system-like antiviral immunity in shrimp. The present results provide the first evidence to show that Cyclophilin A can modulate NLR-mediated cytokine-like antiviral immunity in animals. These findings shed light on the roles of NLRs in regulating host innate immunity, contributing valuable insights into this area of study.

## Author summary

Innate immune recognition is the jewel in the field of innate immunity. The discovery of different pattern recognition receptors, such as Toll-like receptors, has continually led to new breakthroughs in the field of innate immunity. As important intracellular pattern recognition receptors, NLRs have been star molecules since their discovery. However, the mechanisms of action of NLRs in invertebrates still lack in-depth studies. In this study, we identified an NLRC-like molecule, LvNLRC, in the crustacean *Litopenaeus vannamei* and found that it can recognize DNA virus analogs and participate in the viral infection process by regulating the STING-mediated interferon-like pathway. Notably, the shrimp Cyclophilin A plays a significant regulatory role in the above process through direct interaction with LvNLRC. This regulatory mechanism is the first report in the field of NLR immune regulation. The study identifies a previously uncharacterized viral pattern recognition receptor in crustaceans, and elucidates a new mechanism for modulating NLR immune functions. These findings have a significant value in the research field of antiviral immunity in crustaceans and are even impactful in the study of NLR immunology.

## Introduction

Host recognition and the triggered subsequent immune responses play crucial roles in defending against viral infection. The immune responses include the detection of viral components by pattern recognition receptors (PRRs) and the activation of immune cells to destroy virus and infected cells. Toll-like receptors (TLRs), RIG-I-like receptors (RLRs), NOD-like receptors (NLRs), and cyclic GMP-AMP synthase (cGAS) are the classical PRRs acting as virus sensors [1–3]. Antiviral immune signaling pathways triggered by these PRRs include the production of interferons (IFNs) and pro-inflammatory cytokines, activation of NF-κB and IRF transcription factors, induction of antiviral genes such as interferon-stimulated genes (ISGs), activation of phagocytosis, and initiation of apoptosis in infected cells [4]. These processes play crucial roles in orchestrating the host immune response against viral infections.

The well-studied host antiviral immune responses in crustacean are the production of antiviral effectors and induction of phagocytosis. Recognition of viral envelope proteins by Tolls could mediate the production of antimicrobial peptides (AMPs) with antiviral function, such as anti-lipopolysaccharide factor and lysozyme [5,6]. In *Marsupenaeus japonicus*, a scavenger receptor C mediates phagocytosis of WSSV and inhibits viral proliferation in host cells [7]. Invertebrates are considered lack of IFN-mediated antiviral immunity. However, an antiviral cytokine-like molecule, Vago, provides similar antiviral function by activating the JAK-STAT pathway in arthropods [8–11]. Shrimp DDX41 and Toll3 act as WSSV sensors and induce Vago expression through STING-IRF and NF-κB signaling pathways, respectively [10,12–14]. In addition, the shrimp MD-2 recognizes a lipid component of WSSV, cholesta-3,5-diene, and activates the Dorsal-dependent Vago production [12]. Understanding how the host recognizes viruses and subsequently activates antiviral immune signaling pathway are crucial for deepening our knowledge of innate immune defense against virus infection. The reported PRRs recognizing WSSV mainly initiate immune responses by detecting viral envelope proteins or lipid components of the viral envelope [15], while the recognition of viral nucleic acids within host cells is still less investigated.

NLRs are a class of intracellularly classical PRRs in vertebrates, which play important roles in the innate immune responses, including microbial defense, pyroptosis, the activation of pro-inflammatory cytokines such as IL-1β, and the regulation of antiviral immunity [16]. Notably, vertebrates NLRs play an important role in regulation of type I IFN-mediated antiviral immunity [17]. Although the domain composition in invertebrate NLRs is quite different from that in vertebrate NLRs, they also play a significant role in invertebrate innate immune system [18,19]. Previously, we found that one NLR-like genes, LvNLRPL2, participates in WSSV infection in *Litopenaeus vannamei*, whereas it lacks the C-terminal LRR domain, which contributes to PAMPs recognition [20]. Here, one NLR protein containing the LRRs domain was identified in *L. vannamei* and designated as LvNLRC based on phylogenetic analysis. LvNLRC could recognize viral DNA and regulate the expression of shrimp Vago, and this regulation is probably achieved through interaction with Cyclophilin A. The results provide new insights into the recognition and regulation of host antiviral immunity.

## Results

### LvNLRC has similar domain composition to the mammalian NLRC family

**Fig 1:**
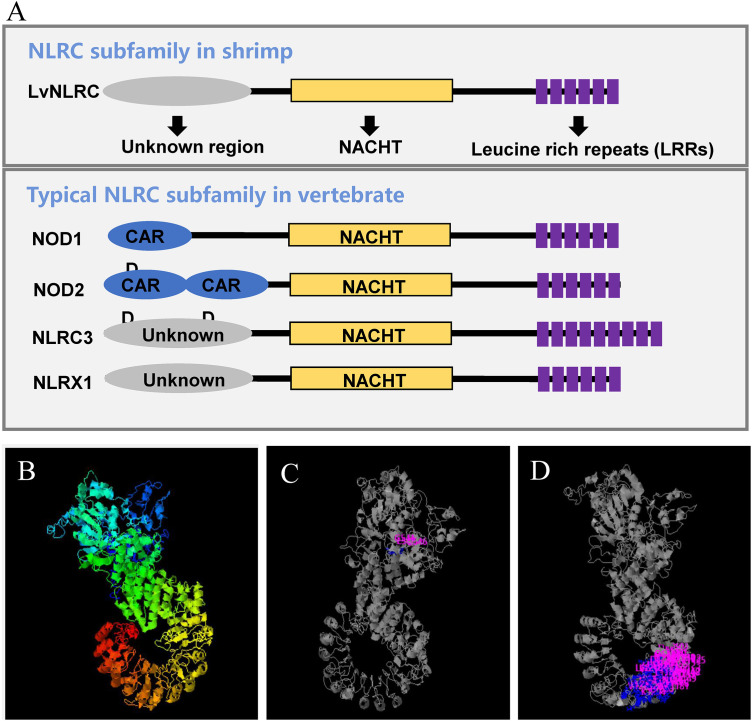
The domain composition of LvNLRC. **(A)** Schematic diagram of the predicted domains of LvNLRC. There are three predicted regions in LvNLRC, including an N-terminal region of unknown function (M1-P349), a central NACHT domain (G350-R750), and the C-terminal LRRs domain in series (L891-L1485). The C-terminal LRRs domain has a typical horseshoe structure. **(B)** The predicted 3D structure of LvNLRC. **(C-D)** The putative ligand-binding sites (the pink and blue position) in central NACHT domain (C) and the horseshoe LRRs domain **(D)**.

**Fig 2:**
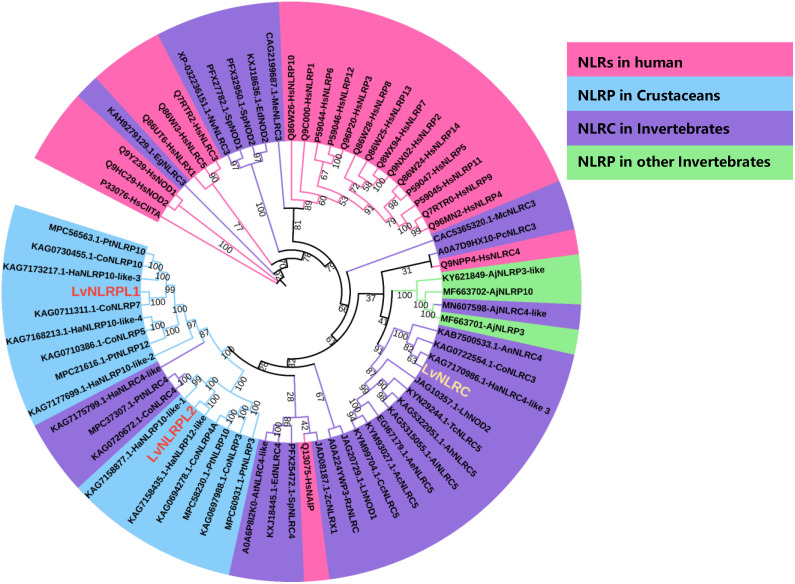
Phylogenetic analysis of LvNLRC based on its full-length amino acid sequence. Amino acid sequences of NLRs or NLR-like genes from different species, including *Homo sapiens*, *Daphnia pulex*, *Eurytemora affinis*, *Hyalella Azteca*, *Armadillidium nasatum*, *Chionoecetes opilio*, *Portunus trituberculatus*, *Homarus americanus*, *Trachymyrmex cornetzi*, *Acromyrmex insinuator*, *Acromyrmex heyeri*, *Acromyrmex echinatior*, *Acromyrmex charruanus*, *Cyphomyrmex costatus*, *Apostichopus japonicus*, *Actinia tenebrosa*, *Exaiptasia diaphana*, *Stylophora pistillata*, and *Nematostella vectensis*, were obtained from GenBank or Uniprot databases. The GenBank or Uniprot accession number of each sequence was shown in each branch. The numbers on the phylogenetic tree represent the bootstrap value.

The total length of LvNLRC open reading frame is 4458 bp, and its encoded protein containes 1485 amino acids. The amino acid sequence of LvNLRC contains an N-terminal region of unknown function (M1-P349), a central NACHT domain (G350-R750), and the LRRs domain in series (L891-L1485) (Fig 1A). The 3D structural model of LvNLRC predicted by I-TASSER has a typical horseshoe structure at the C-terminal LRRs domain (Fig 1B) and multiple putative ligand-binding sites at different positions (Fig 1C and 1D), of which 48 sites are densely distributed in the N-terminal region of the LRRs domain (Fig 1D). This suggests that the LRRs domain of LvNLRC most likely has a ligand-binding function. Phylogenetic analysis showed that LvNLRC clustered together with the NLRC subfamily of several invertebrate species and then clustered with human NLRC family, but was distant from the LvNLRPL1, LvNLRPL2, and other crustacean NLRP-like proteins (Fig 2). Therefore, LvNLRC shows relatively high similarity to that of the NLRC subfamily proteins.

### LvNLRC participates in WSSV infection

**Fig 3:**
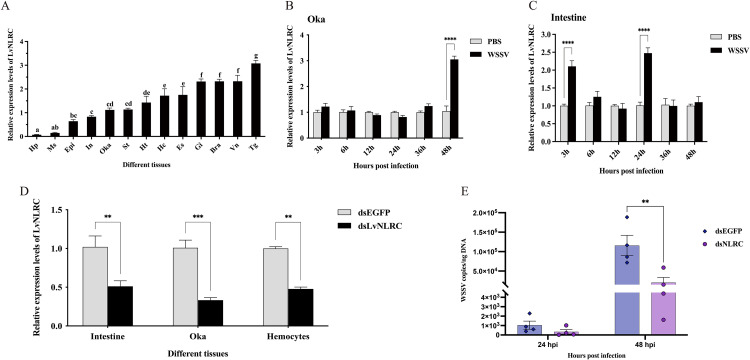
Tissue distribution and the responses of *LvNLRC* to WSSV infection. **(A)** The Tissue distribution of *LvNLRC*. Ordinary one-way ANOVA was performed for statistical analysis. **(B-C)** Expression changes of *LvNLRC* at different time points after WSSV infection in Oka (B) and intestine **(C)**. **(D)** The interference efficiency of dsLvNLRC in intestine, Oka and hemocytes. **(E)** The WSSV copies of *LvNLRC*-knockdown shrimp at 24 hpi and 48 hpi. dsEGFP, the control group injected with EGFP dsRNA and infected with WSSV. dsLvNLRC, the experimental group injected with *LvNLRC* dsRNA and infected with WSSV. Abbreviation interpretation in **(A)**, Hp, hepatopancreas; Ms, muscle; Epi, epithelial; In, intestine; Oka, lymphoid organ; St, stomach; Ht, heart; Hc, hemocytes; Es, eyestalk; Gi, gill; Bra, brain; Vn, ventral nerve cord; Tg, thoracic ganglia. Two-way ANOVA was performed for statistical analysis of **(B)**(C)(D)(E). ^*^P < 0.05; ^**^P < 0.01; ^***^P < 0.001; ^****^P < 0.0001.

To explore whether *LvNLRC* is involved in the innate immune response, we first examined the transcriptional level of *LvNLRC* among tissues and in response to WSSV infection. *LvNLRC* was widely detected in all tested tissues (Fig 3A). *LvNLRC* was significantly up-regulated in Oka by 2.9-fold at 48 h post infection (hpi, Fig 3B). In contrast, *LvNLRC* was up-regulated in intestine at the early and mid-stages of WSSV infection, by 2.1-fold at 3 hpi and 2.5-fold at 24 hpi (Fig 3C). Subsequently, we conducted WSSV infection on shrimp after knocking down *LvNLRC* to further investigate its role in the viral infection process. Based on tissue distribution and pathogen infection responses, we assessed the interference efficiency of dsLvNLRC in immune tissues such as intestine, Oka, and hemocytes after 48 h of dsRNA interference, which were 50%, 67%, and 52%, respectively (Fig 3D). At 24 hpi, both the dsLvNLRC experimental group and the dsEGFP control group exhibited lower WSSV copy numbers in shrimp. At 48 hpi, the viral copy number in *LvNLRC*-knocked down shrimp was significantly lower than those in the dsEGFP control group (Fig 3E). These findings suggest that knockdown of the *LvNLRC* gene can significantly inhibit the proliferation of WSSV in shrimp, indicating that *LvNLRC* may have an inhibitory effect on the antiviral immune pathway.

### LvNLRC recognizes poly(dA:dT) and regulates STING mediated antiviral signaling pathway

**Fig 4:**
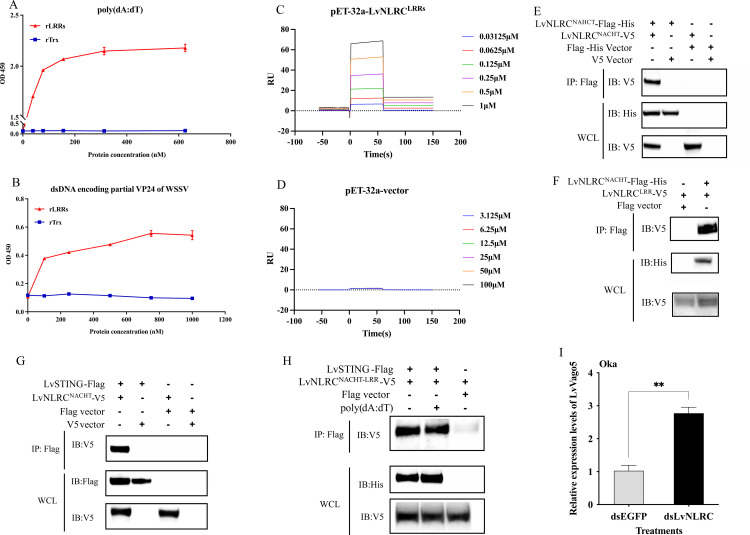
Molecular mechanism of LvNLRC in antiviral immune response. **(A)** The binding ability of rLRRs of LvNLRC to poly(dA:dT) detected by ELISA. **(B)** The binding ability of rLRRs of LvNLRC to dsDNA encoding partial VP24 of WSSV. The assay was detected by ELISA. **(C)** The SPR (surface-plasmon resonance) kinetic fitting of 32a-LvNLRC-LRRs protein binding to poly(dA:dT). The interaction dynamics between 32a-LvNLRC-LRRs and poly(dA:dT) across a concentration gradient from 0.03125 μM to 1 μM. The molecular weight of poly(dA:dT) is calculated according to the molecular weight of the monomer (dA + dT), because there is no fixed molecular weight of poly(dA:dT). **(D)** The negative rTrx control expressed by the empty pET-32a-vector plasmid of the SPR assay across a higher concentration gradient from 3.125 μM to 100 μM. **(E)** Co-immunoprecipitation analysis of the homo-dimerization of LvNLRC. **(F)** Co-immunoprecipitation analysis of the interaction between the NACHT domain and LRRs domain of LvNLRC. **(G)** Co-immunoprecipitation analysis of the interaction between the LvNLRC-NACHT domain and LvSTING. **(H)** The effect of poly(dA:dT) on the association between LvNLRC^NACHT-LRR^ and LvSTING. **(I)** Effect of LvNLRC knockdown on LvVago5 expression level in Oka. LvNLRC^NACHT^ is the NACHT domain of LvNLRC. LvNLRC^LRR^ is the LRRs domain of LvNLRC. Anti-flag-coupled magnetic beads were used for co-immunoprecipitation to detect V5 tagged prey proteins in Co-IP samples by Anti-V5 antibodies. WCL, whole cell lysate. The Flag tagged protein carries His tag and is detected with anti-His antibodies.

As LvNLRC has a typical horseshoe structure at the C-terminal LRRs domain and multiple putative ligand-binding sites (Fig 1B-1D), we conducted the binding assay of the LvNLRC-LRRs with DNA viral mimics. LvNLRC-LRRs could bind to the DNA viral mimics poly(dA:dT) (Fig 4A), indicating the possible ligand binding ability of LRRs to viral nucleic acids. Furthermore, the binding ability of LvNLRC-LRRs to WSSV viral nucleic acids were also conducted by ELISA. As expected, the results showed that LvNLRC-LRRs do has the ability to recognize a DNA sequence encoding partial VP24 of WSSV (Fig 4B). These data demonstrate that LvNLRC-LRRs can recognize viral nucleic acids. To further verify this point, we employed the Surface Plasmon Resonance (SPR) method to test the association of LvNLRC-LRRs to poly(dA:dT). The SPR result clearly demonstrates a definite interaction between LvNLRC-LRRs and poly(dA:dT) in a dose-dependent manner with a dissociation constant about 0.43 μM (Fig 4C), while the control protein expressing by the empty plasmid did not bind to poly(dA:dT) even at very high concentrations (Fig 4D). Next, we explored whether LvNLRC had an intramolecular mechanism similar to the activation process of mammalian NLRC. The results of co-immunoprecipitation showed that the NACHT domain in LvNLRC could at least dimerize with itself (Fig 4E). Here, we used anti-His tag antibody to detect Flag-tagged proteins fused with His tag, as explained in the CoIP section of the Materials and Methods. In addition, NACHT can also interact with LvNLRC-LRRs (Fig 4F), indicating that LvNLRC may maintain an autoinhibitory state in the normal state of cells. The binding ability of LvNLRC to viral nucleic acid and the intramolecular interaction mechanism remind us of the regulatory function of mammalian NLRC3 on STING signaling pathway in response to viral infection. Further studies showed that the NACHT domain could indeed interact with LvSTING (Fig 4G). However, in the presence of poly(dA:dT), the interaction between NACHT and LvSTING was not weakened (Fig 4H). Nevertheless, knockdown of *LvNLRC* could increase the expression level of the IFN analogue *LvVago5* in Oka (Fig 4I), which suggests that there might also be other mechanisms involved in the regulation of LvSTING by LvNLRC in shrimp.

### LvCypA interacts with LvNLRC and regulates WSSV infection and *LvVago5* expression

**Fig 5:**
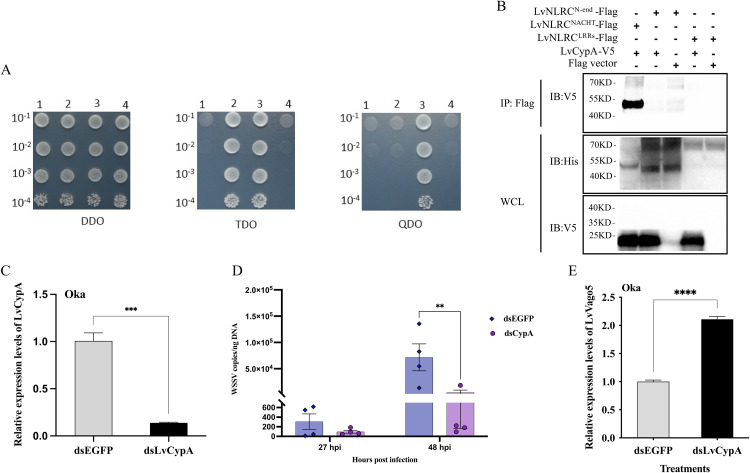
Screening of LvNLRC interacting molecule and its immune responses during WSSV infection in vivo. **(A)** The interaction of LvCypA with full-length LvNLRC verified by yeast two-hybrid. Line1, self-activation test group, Y2H [PGBKT7-A+ PGADT7]. Line 2, experimental group, Y2H [PGBKT7-A+ PGADT7-B]. Line 3, positive control group, Y2H [pGBKT7-53 + pGADT7-T]. Line 4, negative control group, Y2H [pGBKT7-lam + pGADT7-T]. The vertical axis represents the dilution ratio of the spot strain. **(B)** Co-immunoprecipitation of LvCypA with the three domains (N-terminal, NACHT and LRRs) of LvNLRC. LvNLRC^N-end^ is the N-terminal domain of LvNLRC. LvNLRC^NACHT^ is the NACHT domain of LvNLRC. LvNLRC^LRR^ is the LRRs domain of LvNLRC. Anti-flag-coupled magnetic beads were used for co-immunoprecipitation to detect V5 tagged prey proteins in Co-IP samples by Anti-V5 antibodies. WCL, whole cell lysate. The Flag tagged protein carries His tag and is detected with anti-His antibodies. **(C)** Knockdown of *LvCypA* by dsRNA. Unpaired t-test was performed for statistical analysis. **(D)** The WSSV copies of *LvCypA*-knockdown shrimp at 27 hpi and 48 hpi. dsEGFP, the control group injected with EGFP dsRNA and infected with WSSV. dsLvCypA, the experimental group injected with *LvCypA* dsRNA and infected with WSSV. In each treatment, the horizontal line represents the median quartile distance and the dot represents the number of WSSV copies (copies/ng DNA) in each parallel. Two-way ANOVA was performed for statistical analysis. **(E)** Effect of *LvCypA* knockdown on *LvVago5* expression level in Oka. Unpaired t-test was performed for statistical analysis. ^*^P < 0.05; ^**^P < 0.01; ^***^P < 0.001; ^****^P < 0.0001;.

We therefore screened the yeast two-hybrid cDNA library of shrimp for additional molecules that might be regulated by LvNLRC (S1 Table). One of the screening proteins was LvCypA, whose homologous gene plays an important role in mammalian antiviral immunity. However, no correlation between LvCypA and NLRs was reported. Yeast two-hybrid analysis showed that LvCypA could interact with the full-length LvNLRC protein (Fig 5A). Through segmented expression of the three LvNLRC domains in Sf9 cells and co-immunoprecipitation analysis with LvCypA, we found that LvCypA only interacted with the NACHT domain of LvNLRC (Fig 5B). We then investigated the function of LvCypA in the antiviral immunity of shrimp. *In vivo* WSSV copy number was significantly lower in *LvCypA* knockdown shrimp than the control at 48 hpi (Fig 5C and 5D). In addition, knockdown of *LvCypA* in shrimp also up-regulated the expression of *LvVago5* in Oka (Fig 5E), indicating that the presence of LvCypA also affected the activation of interferon related antiviral immune signaling pathway in shrimp.

### The interaction between LvCypA and LvNLRC is affected by poly(dA:dT)

**Fig 6:**
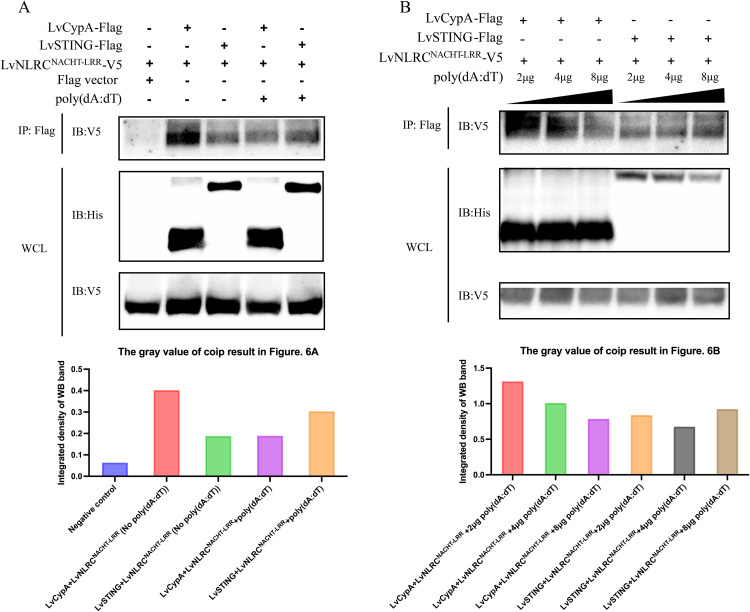
The effect of poly(dA:dT) on the association between LvCypA and LvSTING. **(A)** The interaction between LvCypA and LvNLRC^NACHT-LRR^. **(B)** Effects of different doses of poly(dA:dT) on the interaction between LvCypA and LvSTING, LvNLRC and LvSTING.

The results above suggest that LvCypA not only interacts with the NACHT domain of LvNLRC, but also exhibits a very similar function to LvNLRC during antiviral immune response. Since the presence of poly(dA:dT) has no effect on the interaction of LvNLRC with LvSTING, is it possible that it interferes with the interaction of LvNLRC with LvCypA? Co-immunoprecipitation analysis showed that LvCypA also interacted with the NACHT-LRR truncated protein of LvNLRC (Fig 6A). After adding poly(dA:dT) at different doses, the interaction between LvSTING and NACHT-LRR remained stable (Fig 6B). Unexpectedly, the CoIP band became lighter with increasing doses of poly(dA:dT) in the interaction between LvCypA and NACHT-LRR (Fig 6B). This implies that poly(dA:dT) somehow interferes with the association between LvCypA and NACHT-LRR.

## Discussion

Most of the mammalian NLRs identified so far that participate in viral immune responses act as negative regulators in innate immunity. These include NLRX1, NLRC3, and NLRC5 from the NLRC subfamily, and NLRP2, NLRP4, NLRP6, NLRP11, NLRP12, and NLRP14 from the NLRP subfamily [21]. These NLRs usually inhibit the transduction of antiviral signals by competitively binding to TLRs, RLRs, and downstream regulatory proteins of the cGAS-STING pathway, or by ubiquitinating and degrading these target proteins [22–25]. However, these NLRs do not seem to be used by viruses to escape host immune responses; instead, they function as switches for antiviral immune signaling pathways, strictly and precisely regulating the initiation and effector molecule transcription of antiviral immunity, which prevent excessive inflammatory reactions that could lead to autoimmune diseases [21,26].

Although LvNLRC does not possess the N-terminal CARD domain, it is more similar to the structural composition of NLRC3 and NLRX1 [16,27]. Except for the N-terminal leader sequence of NLRX1 responsible for mitochondrial localization, the regulatory mechanisms of the antiviral signaling pathway by NLRC3 and NLRX1 primarily involve the NACHT and LRRs domains, with little involvement of the N-terminal domain functions [28–30]. Interestingly, both NLRC3 and NLRX1 can hijack STING through their NACHT domain to inhibit the transcriptional activation of type I interferon genes regulated by IRF3 [28,29]. In shrimp, a homologous molecule of STING and the downstream IRF-Vago-JAK/STAT signaling pathway also participate in host antiviral immunity [5,10,31]. As the N-terminal of LvNLRC does not contain a mitochondrial targeting sequence similar to that of NLRX1, and WSSV is a dsDNA virus, we speculate that LvNLRC may have an antiviral immune regulatory function similar to that of NLRC3.

Deletion of mammalian NLRC3 increases the expression of DNA virus-induced IFN-I and cytokines, leading to significantly reduced viral load and disease incidence [32]. This function of NLRC3 is achieved by binding to STING under healthy conditions of the body, and leading to a conformational change of the NACHT domain and enhancement of its ATPase activity upon recognition and binding of viral dsDNA by its LRRs domain, which promotes the release of STING from the NACHT domain and activation of IFN-I transcription [21]. Similarly, the LRRs domain of LvNLRC can recognize the DNA viral nucleic acid mimic poly(dA:dT), and its NACHT domain can directly interact with LvSTING. Moreover, knockdown of *LvNLRC* upregulates the expression of *LvVago5* and inhibits the proliferation of WSSV. Thus, LvNLRC may act as a cytoplasmic sensor for viral nucleic acids, competing with the downstream effector proteins of LvSTING for binding to LvSTING, inhibiting the production of LvVago5, and realizing a negative regulatory role in the antiviral response.

Different from the regulatory mechanism in vertebrates, the presence of poly(dA:dT) does not weaken the interaction between NACHT and LvSTING, which indicates different regulatory mechanisms of NLR-STING interaction between shrimp and vertebrates. We further found that the NACHT domain of LvNLRC can interact with LvCypA, which has never been reported in studies related to NLRs. The mammalian CypA plays multiple roles in promoting or inhibiting viral infection depending on the type of virus and host cell [33]. CypA can stabilize the viral life cycle by interacting with viral proteins, promoting viral proliferation, or by interacting with multiple molecules in the RIG-I-MAVS signaling pathway to promote the production of IFN-I or inflammatory factors, thereby exerting antiviral immune functions [34]. In the present study, knockdown of *LvCypA* up-regulated the expression of *LvVago5* and inhibited viral proliferation, indicating that the presence of *LvCypA* promotes viral infection, which is different from the regulatory role of CypA on interferons in mammals. Additionally, multiple sequence alignments revealed that the NACHT domains of LvNLRC and other STING-binding NLRs, such as HsNLRC3 and HsNLRX1, contain multiple conserved proline residue sites. As a peptide proline isomerase, CypA can promote protein folding by catalyzing the peptide bond on proline residues from trans to cis conformation [33]. Therefore, LvCypA might target the conserved proline residue sites in the NACHT domain of LvNLRC to alter its conformation.

**Fig 7:**
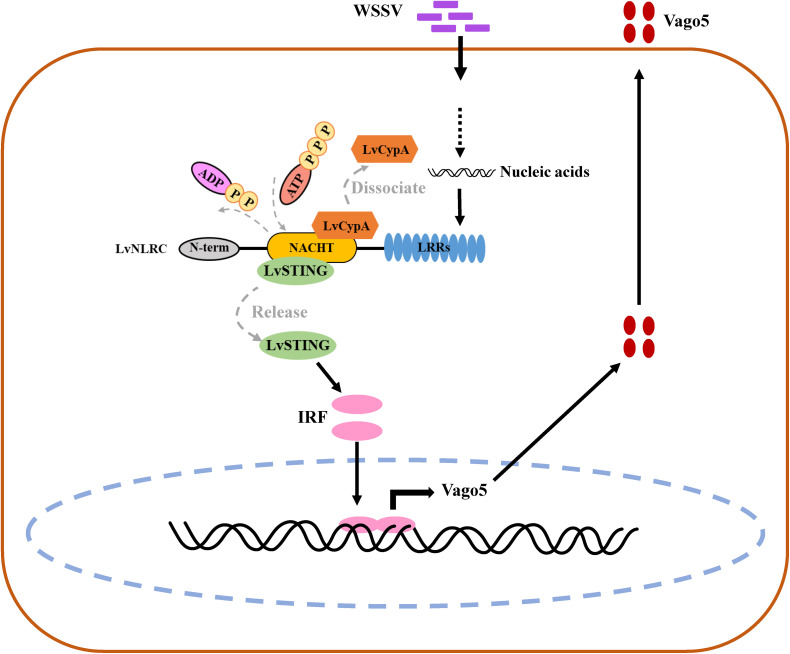
Working model for the LvNLRC-mediated WSSV recognition and activation of Vago5 expression. Normally in healthy shrimp, LvNLRC functions as a switch in antiviral immune signaling pathways rather than an immune escape tool for WSSV. Its NACHT domain competitively binds to STING, inhibiting the transduction of antiviral signals. LvCypA is also attached to the NACHT domain, and their interaction may inhibit the ATPase activity of NACHT domain, thus impeding LvSTING release and downstream antiviral signaling pathway activation. Upon WSSV infection, some viral particles enter the autophagy-lysosome pathway and the viral genome DNA might be released after autolysosome degradation [39]. The LRRs domain of LvNLRC can recognize the viral DNA nucleic acid. This recognition might trigger LvCypA activity. Consequently, LvCypA dissociates from the NACHT domain, which leads to the dissociation of LvSTING. This activates the downstream IRF-Vago signaling pathway, increasing the production of LvVago5 to participate in host antiviral immunity.

Considering that both LvCypA and LvSTING can bind to the NACHT domain of LvNLRC, and the release of STING requires the enhanced ATPase activity of NACHT, we speculate that the interaction between LvCypA and the NACHT domain may inhibit its ATPase activity, thereby impeding the release of STING and the activation of its downstream antiviral signaling pathway. As proposed in the working model, LvCypA dissociates from the NACHT domain upon WSSV infection, and LvNLRC spontaneously transitions to the trans conformation, exposing the ATP binding pocket and catalytic pocket of ATPase, thereby enhancing the ATPase activity, leading to the dissociation of LvSTING (Fig 7). Notably, some CypA inhibitors developed for mammals exhibit broad-spectrum antiviral effects and can effectively prevent the emergence of pathogen resistance [35]. Given the high sequence similarity, up to 76% between LvCypA and human CypA, further investigation of the antiviral immune mechanism of LvNLRC-LvCypA may provide a theoretical basis for developing new methods to control shrimp viruses.

The present study not only reveals the important function and regulatory mechanism of LvNLRC in antiviral immunity of crustacean, but also reports for the first time a novel interacting protein of NLR, CypA, that participates in regulating the interferon system-like antiviral pathway mediated by LvNLRC. The results provide significant clues regarding the role of invertebrate NLRs in innate immunity and enrich our understanding of the diversity and functionality of crustacean pattern recognition receptors, potentially providing a scientific and theoretical basis for effectively preventing and controlling shrimp diseases.

## Materials and methods

### Sequence analysis

The sequences annotated as NLRs were screened from the *Litopenaeus vannamei* transcriptome data [36], and further aligned and verified in the NCBI blastx database (https://blast.ncbi.nlm.nih.gov/Blast.cgi) to select those with the NLRs conserved domains NACHT. NCBI ORF finder (https://www.ncbi.nlm.nih.gov/orffinder/) was used to predict the open reading frames (ORFs) of these sequences and deduce their amino acid sequences. Domain prediction of the deduced amino acid sequences was performed by SMART (http://smart.embl.de/) and InterPro (http://www.ebi.ac.uk/interpro/). Amino acid sequences of NLRs or NLR-like genes of different species were obtained from GenBank or Uniprot databases. The GenBank or Uniprot accession number of each sequence was shown in each branch. The sequence similarity of all sequences was aligned by MUSCLE in MAGA11 software. The phylogenetic tree was constructed based on IQ-Tree Web Server (http://iqtree.cibiv.univie.ac.at/) with VT + F + G4 as the best-fit model [37]. The branch credibility was tested by 1000 bootstrap tests. The 3D protein structure and ligand binding sites of LvNLRC full-length amino acid sequence were predicted through I-TASSER website (https://zhanggroup.org//I-TASSER/).

### Experimental animals, tissue collection and WSSV infection

Before experiments, the experimental animal, *L. vannamei*, were acclimated for 3 days in the filtered clean seawater, which was maintained aerated at a temperature of 25 ± 1 °C. The shrimp were fed with commercial baits twice a day.

Five healthy adult shrimp were randomly picked for tissue distribution detection, with an average body weight of 9.5 ± 0.8 g. The tested tissues included hemocytes, gill, lymphoid organ (Oka), hepatopancreas, heart, stomach, thoracic ganglia, ventral nerve cord, brain, epidermis, eyestalk, intestine and muscle. Hemolymph was collected from the ventral sinus located at the ﬁrst abdominal segment of shrimp using a sterile 2.5-ml syringe that had been drawn with sterile anticoagulant at a volume ratio of approximately 1:1, and immediately mixed. The formulation of anticoagulant-modified Alsever’s solution (pH 7.4) include 115 mM glucose, 27 mM sodium citrate, 336 mM NaCl and 9 mM EDTA•Na_2_•2H_2_O. The hemocytes were collected from the hemolymph by centrifugation at 1000 g for 8 min at 4 °C, and the supernatant was removed. Other tissues were dissected directly and all tissues were snap-frozen in liquid nitrogen for subsequent total RNA extraction, cDNA synthesis and RT-qPCR analysis.

Healthy adult shrimp were randomly picked for WSSV challenge experiments, with an average weight similar to those for the tissue distribution experiment. The stock WSSV solution was diluted on ice using sterile PBS, and a dosage of 10^3^ copies per shrimp was administered with a volume of 10 μL. Similar sized shrimp injected with the same volume of PBS were used as the control. Tissue samples were collected at 3 h, 6 h, 12 h, 24 h, 36 h and 48 h post WSSV infection (hpi) for total RNA extraction, cDNA synthesis and RT-qPCR analysis. Three replicates were set up for each time point, with five shrimp per replicate.

### RNA isolation and cDNA synthesis

The above frozen tissues other than hemolymph were firstly ground to powder in liquid nitrogen. The total RNA from all these tissues was extracted using RNAiso Plus Reagent (TaKaRa, Kyoto, Japan) according to the manufacturer’s protocol. Nanodrop 2000 spectrophotometer (Thermo Fisher Scientific, Waltham, MA, USA) were used for assessing the concentration and purity of the total RNA, and its quality verification was then performed by 1% agarose gel electrophoresis. 1 μl total RNA with a concentration of 1 μg/μl was used for cDNA synthesis using the PrimeScript RT Reagent Kit (TaKaRa, Kyoto, Japan) in 20 μl reaction system. According to the manufacturer’s protocol, the genomic DNA in total RNA was removed, and the first strand of cDNA was synthesized by PrimeScript RT enzyme with random primers.

### Synthesis of dsRNA in vitro and RNA interference

Knockdown of LvNLRC and LvCypA was performed with dsRNA-mediated RNA interference (RNAi). Primers with T7 promoters (see S2 Table for primer sequences) were designed to amplify the DNA templates for in vitro transcription of dsRNA targeted to LvNLRC and LvCypA, respectively. Both strands of the DNA template were transcribed *in vitro* to synthesize dsRNA using Transcriptaid T7 High Yield kit (Thermo, USA) according to the manufacturer’s protocol. The DNA template in the transcripts was digested by DNase I. Then the transcripts were purified using a phenol/chloroform mixture. The dsRNA products before and after RNase A digestion were subjected to 1% agarose gel electrophoresis to analyze the quality of dsRNA synthesis.

Different concentrations of dsRNA were administered to healthy adult prawns. Total RNA was extracted from tissues at 48h after dsRNA administration, and the interference efficiency of dsRNA for LvNLRC or LvCypA was detected by RT-qPCR. Shrimp injected with the same dose of EGFP dsRNA (dsEGFP) served as a control. The optimal interference doses of dsLvNLRC and dsLvCypA selected were 0.25 μg/g body weight and 0.6 μg/g body weight, respectively.

### WSSV challenge after RNAi and tissue DNA extraction for virus copies detection

The immune function of LvNLRC during WSSV infection was investigated by WSSV challenge to the shrimp with LvNLRC or LvCypA knocked down. The shrimp were injected with dsRNA according to the optimal RNAi dose selected above, and shrimp injected with dsEGFP was used as the control. Each group was set up in four replicates, with five healthy adult shrimp in each replicate. The average weight of shrimp used for the RNAi of dsLvNLRC or LvCypA was 11.7 ± 1.5 g or 9.1 ± 1.1 g, respectively. WSSV challenge experiments were performed at 48 h after dsRNA injection at a dose of 4 × 10^3^ copies per shrimp for dsLvNLRC interference and 5 × 10^3^ copies per shrimp for dsLvCypA interference. The shrimp pleopods were collected for viral copy number detection at approximately 24 hpi and 48 hpi. The genomic DNA in the pleopods was extracted using a Genomic DNA extraction kit (TIANGEN, Beijing) according to the protocol. The extracted DNA concentration of each sample was measured by Nanodrop 2000 (Thermo Fisher Scientiﬁc, USA).

### Quantitative real time PCR analysis (qRT-PCR)

Gene expression levels and in vivo viral loads were measured by SYBR Green-based RT-qPCR. The RT-qPCR primers for each gene were listed in S2 Table. The amplification efficiency and specificity of each pair of primers were checked by melting curve. The reaction system was prepared using Thunderbird SYBR qPCR Mix (Toyobo, Osaka, Japan), and then was run in the Eppendorf MasterCycler EP Realplex (Eppendorf, Hamburg, Germany) with a specific RT-qPCR program of denaturation at 95 °C for 2 min; 40 cycles of 95 °C for 15 s, annealing temperature for 15 s, and 72 °C for 30 s. For gene expression levels assays, the cDNA template reverse transcribed from total RNA needed to be diluted 40-fold before detection. Relative mRNA levels of target genes were calculated using the formula of 2^−ΔΔCT^ with 18S rRNA as an internal reference. For in vivo viral loads quantification, the copy number of VP28 gene was used to represent the viral loads according to a previous study [38]. Therefore, the VP28 gene was cloned into the pMD19-T vector and a standard curve of Ct values corresponding to known concentrations of VP28 gene could be generated. The Ct values of unknown samples detected by the above RT-qPCR program could be assigned to the standard curve to calculate WSSV loads.

### Construction of recombinant expression plasmid

In-Fusion Snap Assembly Master Mix (TaKaRa, Kyoto, Japan) was used to construct all recombinant expression plasmids. The prokaryotic recombinant expression plasmid was pET32a(+)/LvNLRC^LRRs^, while the eukaryotic recombinant expression plasmids included pDHsp/LvNLRC^N-end^-V5, pDHsp/LvNLRC^NACHT^-Flag-His, pDHsp/LvNLRC^NACHT^-V5, pDHsp/LvNLRC^LRRs^-V5, pDHsp/LvNLRC^NACHT-LRRs^-V5, pDHsp/LvSTING-Flag-His, pDHsp/LvCypA-Flag-His.

LvNLRC^LRRs^ (L891-L1485) was expressed in *E. coli* and used for ligand-binding ELISA assay. The prokaryotic expression vector pET-32a(+) was purchased from Novagen (Merck, Germany) and allowed the expression of the target protein fused with the Trx tag (N-terminal) and His tag (N-terminal and C-terminal). pET-32a(+) was linearized by two restriction enzymes, QuickCut *BamH* I and QuickCut *EcoR* I (TaKaRa, Kyoto, Japan). According to the manufacturer’s protocol of In-Fusion Snap Assembly Master Mix, primers with 15 nt homologous arms were designed to amplify the DNA fragments of LvNLRC^LRRs^ (see S2 Table for primers information). The PCR reaction system was prepared with Premix Ex Taq Hot Start Version (TaKaRa, Kyoto, Japan). The conditions for the PCR program were denaturation at 98 °C for 5 min, 40 cycles of 98 °C for 10 s, annealing at 55 °C for 2 min, and extension at 72 °C for 40 s. and one cycle of extension at 72 °C for 10 min. The insert was ligated to the linearized vector by In-Fusion Enzyme mix according to the manufacturer’s protocol, and then sequencing verification was performed.

The three domains of LvNLRC (LvNLRC^N-end^ from M1 to A300, LvNLRC^NACHT^ from G350 to R750, and LvNLRC^LRRs^ from L891 to L1485), the truncated protein containing both NACHT and LRRs (LvNLRC^NACHT-LRRs^ from G350 to L1400), LvSTING and LvCypA were recombinant expressed in insect Sf9 cells for co-immunoprecipitation analysis. The vectors used for Sf9 cell expression, pDHsp/V5-His vector and pDHsp/Flag-His vector, were generously provided by Professor Lo (Chang et al., 2010). pDHsp/Flag-His vector was used to express the target protein fused with the Flag tag (C-terminal) and His tag (C-terminal), while pDHsp/V5-His vector expressed the target protein fused with the V5 tag (C-terminal) and His tag (C-terminal). To facilitate detection, the His tag in all pDHsp/V5-His vector was removed by inserting stop codons, so the recombinant protein expressed by pDHsp/V5 vector in this paper did not carry His tag. pDHsp/Flag or V5 vectors were linearized by two restriction enzymes, QuickCut *BamH* I and QuickCut *Hind* III. Primers with 15nt homology arms were also designed to amplify the inserts of LvNLRC^N-end^, LvNLRC^NACHT^, LvNLRC^LRRs^, LvNLRC^NACHT-LRRs^, LvSTING and LvCypA (see S2 Table for primers information). All of these fragments were amplified by Premix Ex Taq Hot Start Version, and the extension times of these PCR programs were 60 s, 100 s, 120 s, 200 s, 70 s, and 50 s, respectively. The annealing temperatures are shown in S2 Table. The inserts were ligated to each linearized vector by In-Fusion Enzyme mix according to the manufacturer’s protocol, sequencing verification was performed.

### Induction and purification of prokaryotic recombinant protein

The constructed pET-32a-LvNLRC^LRRs^ recombinant plasmid and the empty pET-32a(+) were separately transformed into *Transetta*(DE3) *E. coli* competent cells (TransGen Biotech, Beijing), and evenly spread on LB solid medium containing ampicillin. The colony PCR products amplified by universal primers (T7 and T7-ter, see S2 Table) detected by 1% agarose gel electrophoresis were screened for positive clones, which then were inoculated into 5 mL LB liquid medium (containing ampicillin) and cultured overnight at 37 °C and 220 rpm. The bacterial culture was re-inoculated at a ratio of 1:100 into 200 mL of LB liquid medium (containing ampicillin), and incubated at 37 °C and 220 rpm for 4–5 hours until the OD_600_ reached 0.4-0.6. Then, 1 mL of the culture was taken as a negative control for protein expression. The remaining bacterial culture was induced at 16 °C and 160 rpm for 24 h after added with IPTG (final concentration, 0.1 mM). 1 mL of IPTG-induced bacterial culture was used to detect protein expression and solubility, and the remaining was separated by centrifugation and stored at -20 °C. The supernatant and inclusion bodies of the bacterial cells before and after IPTG induction were separated through ultrasonic disruption and centrifugation. The rLRRs protein was expressed in the form of inclusion bodies detected by denaturing electrophoresis on SDS-PAGE (8%-16%) gels and Coomassie brilliant blue staining. In contrast, the ELISA negative control protein rTrx-His expressed by empty pET-32a(+) was soluble.

The inclusion bodies rLRRs and soluble rTrx-His were purified using TALON Metal Affinity Resin (TaKaRa, Kyoto, Japan). The recombinant protein in the remaining IPTG-induced bacterial culture was acquired as described above. The inclusion bodies were washed with TALON Metal Affinity Resin Equilibration Buffer (50 mM Sodium phosphate, 300 mM Sodium chloride, pH 7.4) containing 2 M urea, then dissolved in TALON Metal Affinity Resin Equilibration Buffer containing 8M urea, and purified according to the manufacturer’s protocol. The soluble rTrx-His was purified in buffer without urea.

The purified inclusion body sample was then renatured. The purified rLRRs product was dialyzed sequentially in the refolding buffer (50 mM Tris, 50 mM NaCl, 1 mM EDTA, 2 mM Glutathione reduced, 0.2 mM Glutathione oxidized, 10% glycerin, 1% Glycine) containing 6 M, 4 M, 3 M, 2 M, 1 M, and 0 M urea respectively, and then transferred to Tris-HCl buffer (50 mM Tris, 50 mM NaCl, pH 8.0) for three rounds of dialysis, each for 8 hours, with continuous stirring using a magnetic stirrer. Meanwhile, the purified rTrx-His was dialyzed three times in Tris-HCl buffer as well. The purified and renatured samples were detected by SDS-PAGE denaturing electrophoresis and Coomassie brilliant blue staining. Finally, we obtained the relatively clean target protein and rTrx-His band. The protein concentrations were determined by a BCA Protein Quantification Kit (Vazyme, China).

### Enzyme linked immunosorbent assay (ELISA)

Investigating the binding ability of rLRRs to poly(dA:dT) and VP24-DNA by ELISA. The purified and refolded protein solutions obtained above were concentrated to a suitable concentration and then filtered through a 0.22 µm Millex-GP Syringe Filter Unit (MilliporeSigma, America). Poly(dA:dT) (InvivoGen, France) was dissolved in the carbonate buffer (15 mM Na_2_CO_3_, 35 mM NaHCO_3_, pH 9.6) to a final concentration of approximately 10 μg/100 μL, and then added to each well of a 96-well ELISA plate (NEST, Wuxi, China) at a volume of 100 μL and incubated at 4 °C for 18 hours to coat the substrate poly(dA:dT) on the ELISA plate. Subsequently, the ELISA plate coated with poly(dA:dT) was washed 3 times with PBST, and blocked with 3% BSA (prepared in PBS) with 200 μl per well at 37 °C for 1 h. A 23 bp fragment encoding WSSV VP24 (VP24-DNA, AATCTAAAGAATTAGAATTGTAT) was synthesized and used for coating the ELISA plate as described above. Meanwhile, the rLRRs was diluted gradient with Tris-HCl (pH 8.0) to the final concentrations of 0 nM, 39 nM, 78 nM, 156 nM, 313 nM, 625 nM, and 1250 nM, respectively. After blocking and washed with PBST, the ELISA plate was added with 100 μl of each concentration of rLRRs in each well with 3 replicates and incubated at room temperature for 3 hours. The pET-32a(+) empty tag rTrx-His was used as a control at the same molar concentration. Followed with target protein incubation, the ELISA plates were incubated successively with anti-His tag antibody (ABclonal, Wuhan, China) and HRP-linked secondary antibody (Cell Signaling Technology, USA) at 37 °C for 1 h, respectively. Both primary and secondary antibodies were diluted 1/1000-fold in PBS containing 0.1 mg/mL BSA, and 100 μL was added to each well. Finally, the ELISA plate was thoroughly washed with PBST and then subjected to color development using EL-TMB Chromogenic Reagent kit (Sangon Biotech, Shanghai, China). The absorbance was measured at a wavelength of 450 nm.

### Surface Plasmon Resonance (SPR) analysis

Experiments were performed in at 25 °C on a BIAcore 1K using CM5 sensor chips, and data were analyzed using BIAcore 1K Evaluation software (Cytiva) following the manufacturer’s instruction. In brief, a cell on the CM5 sensor chip was activated with a mixture of 200 μM 1-ethyl-3-(3-dimethylaminopropyl) carbodiimide (EDC, Cytiva) and 50 μM N-hydroxysuccinimide (NHS, Cytiva) at 10 μl/min for 420s. A total of 50 μl of 32a-NLRC-LRR protein by mixing with 180 μl of 10 mM sodium acetate solution, pH 5.0, was then immobilized on the surface of the cell at 10 μl/min for 420s for two repetitive runs. Run the rTrx protein expressing by pET-32a empty vector in the same way as the negative control. The cell was then blocked with 1 M ethanolamine (10 μl/min for 420s). A neighboring aisle that served as a reference was similarly activated and blocked, except that PBS adjusted to pH 5.0 was used for immobilization. Both of the aisle was then equilibrated with PBS. The poly(dA:dT) stock solution was diluted to a series of concentrations in PBS, and was flowed at 30 μl/min for 150 s in each run. For 32a-NLRC-LRR, poly(dA:dT) was across a concentration gradient from 0.03125 μM to 1 μM. And for rTrx protein, poly(dA:dT) was across a higher concentration gradient from 3.125 μM to 100 μM. The molecular weight of poly(dA:dT) is calculated according to the molecular weight of the monomer (dA + dT), because there is no fixed molecular weight of poly(dA:dT). At the end of each flow, cells were regenerated for 5 min with 10 mM glycine-HCl (pH 2.0) solution at 10 μl/ min for 5 min. Data from the sample cell were collected using Biacore Insight (v. 2.0, Cytiva), and were subtracted by those from the reference cell. Association and dissociation constants were obtained by global fitting of the data to a 1:1 Langmuir binding model using BIAcore 1K Evaluation software (Cytiva, Marlborough, MA, USA).

### Explore the regulatory effect of poly(dA:dT) on LvNLRC related signaling pathway

The plasmids combination of pDHsp/LvCypA and pDHsp/LvNLRC-LRRs-V5, and the plasmids combination of pDHsp/LvSTING and pDHsp/LvNLRC-LRRs-V5 were co-transfected into Sf9 cells, respectively. After overnight incubation at 27 °C, Sf9 cells was initiated for protein expression by heat-shock at 42 °C for 30 min. The poly(dA:dT) was transfected into the Sf9 cells at 2 μg, 4 μg and 8 μg through Lipofectamine 3000 transfection reagent at 24 h post heat-shock. After 8 h of poly(dA:dT) treatment, cells were lysed with mild lysis buffer containing 5 mM EDTA, and the supernatant was collected for immunoprecipitation analysis. Both co-immunoprecipitation samples and Input samples were determined by western blot.

### Construction of a L. vannamei cDNA library for yeast two-hybrid screening

Total RNA was extracted from tissues with high expression of LvNLRC, and mRNA was purified from total RNA using Dynabeads Oligo(dT)_25_ (Invitrogen, USA) according to the manufacturer’s instructions. The first strand of cDNA was synthesized using the mRNA as template by SMART MMLV Reverse Transcriptase (TaKaRa, Kyoto, Japan), and then the double-stranded cDNA was amplified using Advantage 2 Polymerase Mix (TaKaRa, Kyoto, Japan). The DNA-RNA heterozygous strands were removed by RNase H. The dsDNA products were fractionated and purified using CHROMA SPIN+TE-400 Columns (TaKaRa, Kyoto, Japan), and then subjected to DNA homogenization. The homogenized dsDNA was inserted into pGADT7-Smal-1/2/3 plasmids by In-Fusion Snap Assembly Master Mix to construct a three-ORF plasmid library for mRNA expression. The constructed plasmid library was transformed into *E. coli*, and the monoclonal colonies were randomly selected for colony PCR reaction and sequencing alignment to determining the positive rate of the inserted fragments in the library.

### Yeast two-hybrid screening for proteins interacting with LvNLRC

The LvNLRC full-length ORF fragment was ligated into the pGBKT7 vector to construct the bait recombinant plasmid. Co-transformation of bait recombinant plasmid and empty prey plasmid into Y2H gold yeast competent cells. The toxicity and autoactivation ability of the bait proteins were detected by observing the growth of the co-transformants on DDO (SD/-Leu/-Trp), TDO (SD/-LEU/-TRP/-HIS) and QDO (SD/-LEU/-TRP/-HIS/-ADE) solid media. The results showed that the bait protein was not toxic to yeast cells and did not occur autoactivation. The bait recombinant plasmid was transformed into Y2H gold competent cells, and positive monoclonal colonies were screened by SD/-Trp solid medium. Y2H gold carrying the LvNLRC bait recombinant plasmid was prepared as yeast competent cells. The cDNA plasmid library of *L. vannamei* was then transformed into the competent cells. Positive monoclonal colonies were selected on TDO/X (SD/-LEU/-TRP/-HIS/ X-a-Gal) solid medium and then seeded on the QDO/X (SD/-LEU/-TRP/-HIS/-ADE/ X-a-Gal) plates for further selection. All positive clones were inoculated in TDO liquid medium for expanded culture, and yeast plasmid extraction kit was used to extract plasmids from each sample. The full-length ORF of the selected prey protein LvCypA was ligated into the pGADT7 vector, and the recombinant plasmid was transformed into Y2H gold competent cells carrying the LvNLRC bait recombinant plasmid. Yeast two-hybrid point-to-point validation of the interaction between LvCypA and LvNLRC was performed again using the method described above.

### Co-Immunoprecipitation (CoIP)

The recombinant pDHsp/V5 vectors or pDHsp/Flag-His vectors with each target gene were transformed into **Trans5*α *E. coli** and cultured in low-salt LB liquid medium (NaCl, 5 g/L) containing zeocin for expansion. Endotoxin-free plasmids were extracted using the Endo-free Plasmid DNA Kit (OMEGA, USA). Resuscitated Sf9 cells can achieve a transfection efficiency suitable for WB detection after 4–5 passages of culture. Sf9 cells which have a good transfection efficiency were plated in 6-well plates (CORNING, USA), with approximately 1.2 × 10^6^ cells in 2 mL of cell medium Sf-900 II SFM (Gibco, USA) per well. The cells were cultured at 27 °C for 24 h before transfection. Different combinations of recombinant plasmids were transfected into Sf9 using Lipofectamine 3000 transfection reagent (Invitrogen, USA) and Opti-MEM I medium (Gibco, USA) according to the manufacturer’s instructions. Two wells were transfected for each plasmid combination. The transfected cells were cultured at 27 °C overnight and then heat-shocked at 42 °C for 30 min to initiate protein expression. After heat shock, the cells were cultured at 27 °C for 24 h for subsequent co-immunoprecipitation analysis. Cells were lysed by mild cell lysis buffer (Beyotime, Shanghai, China) supplemented with protease inhibitor PMSF (1mM) according to the manufacturer’s protocol. The mixture was centrifuged at 4 °C and 12000 rpm for 10 min to remove cell debris and other impurities. A quarter of the supernatant was used as Input to detect the expression of the target protein of each transfected plasmid. The remaining supernatant was incubated with anti-FLAG M2 Magnetic Beads at 4 °C for 2 h with rotation to capture the Flag-tagged proteins and their interacting proteins. After incubation, the magnetic beads were washed thoroughly, and then the proteins on the beads were eluted by boiling. The V5-tagged proteins co-immunoprecipitated with the Flag-tagged proteins were detected by western blot analysis. Since the Flag-tagged protein was also coupled to His tag, which is more easily detected for WB assay, anti-His tag antibody was used to detect the presence of Flag-tagged protein in our CoIP method.

## Supporting information

S1 Table The protein information with potential interaction with LvNLRC screened from the yeast two-hybrid library. (DOCX)

S2 Table Sequence information of primers used in the functional study of LvNLRC. (DOCX)

S1 Information The immunoblot raw files corresponding to Figs 4E, 4F, 4G, 4H, 5B, 6A and 6B. (7Z)
