# Supplementary material for: An invertebrate NLR recognizes viral nucleic acids and balances the antiviral signaling pathway through interaction with STING and Cyclophilin A
Source: PLoS Pathog. 2025 Aug 18;21(8):e1013433. doi: 10.1371/journal.ppat.1013433 (PMC12370184; doi:10.1371/journal.ppat.1013433)
Supplement: S1 Table — (DOCX) [file ppat.1013433.s001.docx]

S1 Table: The protein information with potential interaction with LvNLRC screened from the yeast two-hybrid library

| Gene No. | NCBI ID | Uniprot ID |
| --- | --- | --- |
| 1 | unknown | unknown |
| 2 | [AGY36411.1](https://www.ncbi.nlm.nih.gov/protein/AGY36411.1?report=genbank&log$=protalign&blast_rank=1&RID=XJWM1MPK01N) | U5P265 |
| 3 | [ROT76407.1](https://www.ncbi.nlm.nih.gov/protein/ROT76407.1?report=genbank&log$=protalign&blast_rank=1&RID=XJXXHBT1016) | A0A3R7PM47 |
| 4 | [ROT81909.1](https://www.ncbi.nlm.nih.gov/protein/ROT81909.1?report=genbank&log$=protalign&blast_rank=5&RID=XK619YAC013) | A0A423TZM9 |
| 5 | unknown | unknown |
| 6 | [QJQ72429.1](https://www.ncbi.nlm.nih.gov/protein/QJQ72429.1?report=genbank&log$=protalign&blast_rank=1&RID=XK766KH7013) | A0A6M4DU43 |
| 7 | [ANH58180.1](https://www.ncbi.nlm.nih.gov/protein/ANH58180.1?report=genbank&log$=protalign&blast_rank=1&RID=XK7JJYBE016) | A0A173GPI6 |
| 8 | [CCE46016.1](https://www.ncbi.nlm.nih.gov/protein/CCE46016.1?report=genbank&log$=protalign&blast_rank=2&RID=XK81TJ4U013) | G8BLI9 |
| 9 | [XP_003130681.2](https://www.ncbi.nlm.nih.gov/protein/XP_003130681.2?report=genbank&log$=protalign&blast_rank=1&RID=XK8RA6UF013) | A0A3E1N6K2 |
| 10 | unknown | unknown |
| 11 | [KAB7501160.1](https://www.ncbi.nlm.nih.gov/protein/KAB7501160.1?report=genbank&log$=protalign&blast_rank=4&RID=XKB3MG0A013) |  |
| 12 | [XP_003130681.2](https://www.ncbi.nlm.nih.gov/protein/XP_003130681.2?report=genbank&log$=protalign&blast_rank=1&RID=XKBS9MU3013) | UPI000281A4D5 |
| 13 | unknown | unknown |
| 14 | [ANA78437.1](https://www.ncbi.nlm.nih.gov/protein/ANA78437.1?report=genbank&log$=protalign&blast_rank=1&RID=XKCJWURM013) | unknown |
| 15 | unknown | unknown |
| 16 | [BAH28836.1](https://www.ncbi.nlm.nih.gov/protein/BAH28836.1?report=genbank&log$=protalign&blast_rank=2&RID=XKD715ZR013) | B9ZZQ1 |
| 17 | unknown | unknown |
| 18 | [QJQ72429.1](https://www.ncbi.nlm.nih.gov/protein/QJQ72429.1?report=genbank&log$=protalign&blast_rank=1&RID=XKE5P9CJ013) | A0A6M4DU43 |
| 19 | unknown | unknown |
| 20 | [BAH28836.1](https://www.ncbi.nlm.nih.gov/protein/BAH28836.1?report=genbank&log$=protalign&blast_rank=1&RID=XKES4SVM013) | B9ZZQ1 |
| 21 | [MPC16696.1](https://www.ncbi.nlm.nih.gov/protein/MPC16696.1?report=genbank&log$=protalign&blast_rank=3&RID=XKF4RB3V016) | A0A5B7D618 |
| 22 | unknown | unknown |
| 23 | unknown | unknown |
| 24 | unknown | unknown |
| 25 | [XP_003130681.2](https://www.ncbi.nlm.nih.gov/protein/XP_003130681.2?report=genbank&log$=protalign&blast_rank=1&RID=XKGFN1KU013) | A0A3E1N6K2 |
| 26 | unknown | unknown |
| 27 | [XP_027225835.1](https://www.ncbi.nlm.nih.gov/protein/XP_027225835.1?report=genbank&log$=protalign&blast_rank=1&RID=XKH90F2C013) | A0A129P453 |
| 28 | [XP_003130681.2](https://www.ncbi.nlm.nih.gov/protein/XP_003130681.2?report=genbank&log$=protalign&blast_rank=1&RID=XKHKY1UK016) | unknown |
| 29 | [XP_027234952.1](https://www.ncbi.nlm.nih.gov/protein/XP_027234952.1?report=genbank&log$=protalign&blast_rank=1&RID=XKHY91NC013) | UPI000406D4D3 |
| 30 | [ROT76407.1](https://www.ncbi.nlm.nih.gov/protein/ROT76407.1?report=genbank&log$=protalign&blast_rank=1&RID=XKJA5V83013) | unknown |
