# Supplementary material for: An invertebrate NLR recognizes viral nucleic acids and balances the antiviral signaling pathway through interaction with STING and Cyclophilin A
Source: PLoS Pathog. 2025 Aug 18;21(8):e1013433. doi: 10.1371/journal.ppat.1013433 (PMC12370184; doi:10.1371/journal.ppat.1013433)
Supplement: S2 Table — (DOCX) [file ppat.1013433.s002.docx]

**S2 Table: Sequence information of primers used in the functional study of LvNLRC**

| **Primer Name** | **Primer sequences (5’-3’)** | **Annealing temperature** |
| --- | --- | --- |
| **For bacterial colonies PCR** | |  |
| RV-M | GAGCGGATAACAATTTCACAC | 55 ℃ |
| M13-47 | AGGGTTTTCCCAGTCACG |  |
| pDHsp70-F | TACTTTCAACAAGTCGTTACCGAG | 54 ℃ |
| pDHsp70-R | CATGACAATACAAACTAAGATTTAGTCA |  |
| T7 | TAATACGACTCACTATAGGG |  |
| T7-ter | GCTAGTTATTGCTCAGCGG |  |
| pGADT7-F (T7) | TAATACGACTCACTATAGGGCGAGCGCCGCCATG | 59 ℃ |
| pGADT7-R (ADR) | GTGAACTTGCGGGGTTTTTCAGTATCTACGATT |  |
| **For qRT-PCR** |  |  |
| 18S-F | TATACGCTAGTGGAGCTGGAA | 55℃ |
| 18S-R | GGGGAGGTAGTGACGAAAAAT |  |
| LvNLRC-qF | GGAATGAGGTACTGTCCCTGATA | 55℃ |
| LvNLRC-qR | CAGGTCTTCATACAGGACACCTA |  |
| LvCypA-qF | CAGTTCTTCATCTGCACC | 57℃ |
| LvCypA-qR | TCACCACTTTCCGCTTGC |  |
| LvVago5-qF | CATAGCCAGGCACGAAAG | 52℃ |
| LvVago5-qR | GACCGTCAGCACAAGCAG |  |
| VP28-qF | AAACCTCCGCATTCCTGTGA | 55℃ |
| VP28-qR | TCCGCATCTTCTTCCTTCAT |  |
| **For RNAi** |  |  |
| dsEGFP-F | TAATACGACTCACTATAGGGCAGTGCTTCAGCCGCTACCC | 62℃ |
| dsEGFP-R | TAATACGACTCACTATAGGGAGTTCACCTTGATGCCGTTCTT |  |
| dsLvNLRC-F | TAATACGACTCACTATAGGGTGGCTTGTGGTTTAGATGGC | 62℃ |
| dsLvNLRC-R | TAATACGACTCACTATAGGGAAGGGATGAAAGGCACTTGG |  |
| dsLvCypA-F | TAATACGACTCACTATAGGGATGGGCAATCCCAAAGTCTTTTTCGA | 62℃ |
| dsLvCypA-R | TAATACGACTCACTATAGGGTTACAGCTGGCCGCAGTTGGC |  |
| **For expression plasmid construction** | |  |
| pET32a-LvNLRC^LRRs^-F | GGCTGATATCGGATCCCTAGAGTCTTTTGAGATTCAGGTGA | 55℃ |
| pET32a-LvNLRC^LRRs^-R | GACGGAGCTCGAATTTAGCTCACTCAATCTATCTAATGTG |  |
| pDHsp-LvNLRC^NACHT^-F | GTTACCGAGGAAGCTTATGGGATTTAAGATGATAGTGAGTGAG | 55℃ |
| pDHsp-LvNLRC^NACHT^-R | CTGGACTAGTGGATCCTCGCAAGATCATCAAGCC |  |
| pDHsp- LvNLRC^N-terminal^-F | GTTACCGAGGAAGCTTATGTCCACTGCGTCAGGA | 58℃ |
| pDHsp- LvNLRC^N-terminal^-R | CTGGACTAGTGGATCCAGCCACGTACTTGGGTTC |  |
| pDHsp- LvNLRC^LRRs^-F | GTTACCGAGGAAGCTTATGCTAGAGTCTTTTGAGATTCAGGTGA | 55℃ |
| pDHsp- LvNLRC^LRRs^-R | CTGGACTAGTGGATCCTAGCTCACTCAATCTATCTAATGTG |  |
| pDHsp- LvNLRC^NACHT-LRRs^-F | GTTACCGAGGAAGCTTATGGGATTTAAGATGATAGTGAGTGAGT | 54℃ |
| pDHsp- LvNLRC ^NACHT-LRRs^-R | CTGGACTAGTGGATCCAAGCTTCAAGCCAGCAAGA |  |
| pDHsp-LvCypA-F | GTTACCGAGGAAGCTTATGGGCAATCCCAAAGTCTTTTTCGA | 56℃ |
| pDHsp-LvCypA-R | CTGGACTAGTGGATCCCAGCTGGCCGCAGTTGGC |  |
| pDHsp-LvSTING-F | GTTACCGAGGAAGCTTATGAAGGGAGACGAGCTG | 55℃ |
| pDHsp-LvSTING-R | CTGGACTAGTGGATCCCTTCCGTTCCGTCATTTCGT |  |
| pGBKT7-LvNLRC-F | ATGGCCATGGAGGCCGAATTCATGTCCACTGCGTCAGGAGTG | 60℃ |
| pGBKT7-LvNLRC-R | CCGCTGCAGGTCGACGGATCCTAGCTCACTCAATCTATCTAATGTGGATAT |  |
| pGADT7-LvCypA-F | ACGACGTACCAGATTACGCTCATATGGGCAATCCCAAAGTCTTTTTC | 56℃ |
| pGADT7-LvCypA-R | ATCTGCAGCTCGAGCTCGATGGATCCTTACAGCTGGCCGCAGTTGG |  |

Note: The T7 promoters are indicated by a straight line; the homologous arms in primers for homologous recombination are indicated with a wavy line.。
